# Smart Endoscopy Is Greener Endoscopy: Leveraging Artificial Intelligence and Blockchain Technologies to Drive Sustainability in Digestive Health Care

**DOI:** 10.3390/diagnostics13243625

**Published:** 2023-12-08

**Authors:** Miguel Mascarenhas, Tiago Ribeiro, João Afonso, Francisco Mendes, Pedro Cardoso, Miguel Martins, João Ferreira, Guilherme Macedo

**Affiliations:** 1 Faculty of Medicine, University of Porto, 4200-319 Porto, Portugal; guilhermemacedo59@gmail.com; 2 Precision Medicine Unit, Department of Gastroenterology, Hospital São João, 4200-437 Porto, Portugal; tiagofcribeiro@outlook.com (T.R.); joaoafonso28@gmail.com (J.A.); pedromarilio@gmail.com (P.C.); miguel.pedro96@gmail.com (M.M.); 3 WGO Training Center, 4200-437 Porto, Portugal; 4 Faculty of Engineering, University of Porto, 4200-465 Porto, Portugal; jferreira@fe.up.pt

**Keywords:** artificial intelligence, capsule endoscopy, digestive health care, deep learning, blockchain, convoluted neural networks, greenhouse gases, sustainability, carbon offsetting

## Abstract

The surge in the implementation of artificial intelligence (AI) in recent years has permeated many aspects of our life, and health care is no exception. Whereas this technology can offer clear benefits, some of the problems associated with its use have also been recognised and brought into question, for example, its environmental impact. In a similar fashion, health care also has a significant environmental impact, and it requires a considerable source of greenhouse gases. Whereas efforts are being made to reduce the footprint of AI tools, here, we were specifically interested in how employing AI tools in gastroenterology departments, and in particular in conjunction with capsule endoscopy, can reduce the carbon footprint associated with digestive health care while offering improvements, particularly in terms of diagnostic accuracy. We address the different ways that leveraging AI applications can reduce the carbon footprint associated with all types of capsule endoscopy examinations. Moreover, we contemplate how the incorporation of other technologies, such as blockchain technology, into digestive health care can help ensure the sustainability of this clinical speciality and by extension, health care in general.

## 1. Introduction

One of the most important developments of recent years has been the incorporation of artificial intelligence (AI) into many areas of our daily lives. Whereas IT areas with more commercial goals have understandably taken the lead in this field, this advance is now permeating many other areas where greater control over data management and IT applications may be necessary, such as health care. However, the consequences of healthcare decisions, particularly in relation to disease diagnosis and treatment, in conjunction with the standardization of clinical protocols, imply that comprehensive studies must be carried out prior to the introduction of new technologies and devices in this field. The interest of AI-based tools in health care is reflected by the large number and the growing size of such studies in all areas of health care, focusing on both clinical applications and patient management [1]. Whereas many of these studies have shown the potential benefits of implementing AI tools in terms of diagnostic accuracy and screening improvements, the longer term benefits in relation to patient outcomes remain to be seen [2].

Medical imaging is one clinical area in which AI solutions are being designed as screening and diagnostic tools [3,4,5,6]. Medical imaging is used extensively in gastroenterology as visualization of the gastrointestinal (GI) tract is fundamental to detect and monitor lesions, or changes in this structure. This revolution has been facilitated by the digitalization of different medical imaging techniques and the more widespread use of Electronic Medical Records (EMRs). Machine Learning (ML) and, in particular, Deep Learning (DL) approaches, such as the use of Convoluted Neural Networks (CNNs), have proven interesting in developing tools for the automated analysis of medical images [7,8]. Indeed, an increasing number of CNN-based models are being designed to detect different lesions or conditions in the digestive system through the automated analysis of endoscopy images [9,10,11,12,13,14,15,16]. Moreover, the use of these applications in conjunction with Capsule Endoscopy (CE) is paving the way towards greater automation of a range of endoscopy examinations, including panendoscopy studies [17,18,19,20,21,22].

**Figure 1:**
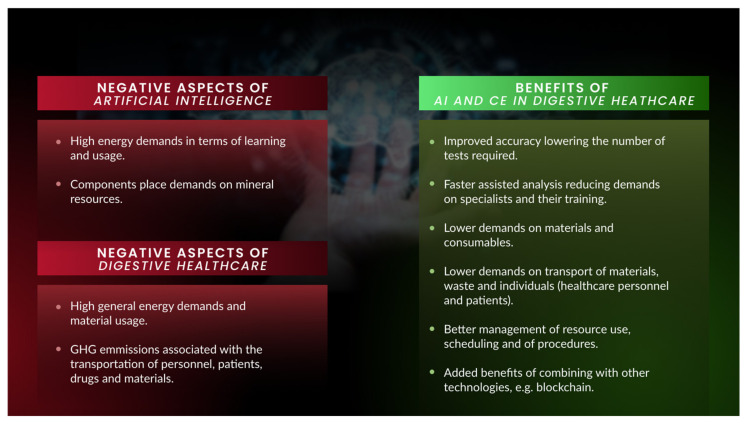
The negative environmental impact of both AI applications and health care in general can be offset, and perhaps overridden, by the benefits that can be gained when applying AI applications in a digestive healthcare setting.

Despite the promise offered by AI-based solutions in the clinic, there has been growing concern in recent years over some of the more negative aspects of this technology. Concerns have been raised for some time regarding the legal and ethical issues associated with the use of AI-based tools [23], particularly regarding aspects such as data protection [24,25,26,27,28]. Moreover, and in accord with the concerns of our society in other areas, the environmental impact of AI-based technologies has begun to be questioned (Figure 1). The implementation of AI tools exerts important demands on energy use, and the equipment required places additional demands on natural resources and their extraction, which has led to serious concerns over the environmental impact and carbon footprint associated with AI applications [29,30]. Indeed, the carbon footprint associated with the development and use of DL applications is undeniably large, augmenting with the use of large datasets, the time such applications are active, the training they need and the computing power they require [31]. Thus, there has been considerable effort of late to try to reduce the carbon footprint associated with the use of AI, and attempts are being made to try to achieve a net carbon balance through the use of greener solutions and “carbon offsetting” [32,33,34]. Significantly, health care is also considered to have an important environmental impact and to contribute significantly to the emission of greenhouse gases (GHGs), with up to 10% of the annual global GHG production attributed to activities within this sphere [23,35,36]. However, health care is a fundamental activity, and it should be delivered universally, with no limitations based on sociodemographic features. Thus, restricting the healthcare activities on the basis environmental impact raises important ethical issues.

In this review, we consider the carbon footprint associated with digestive endoscopy and the use of AI in association with these procedures. (In the preparation of this review article, we retrieved publications from the past 20 years through a PubMed database search using combinations of the search terms: ((Artificial Intelligence[Title]) AND (deep learning[Text])) OR (Machine Learning[Text Word]) OR (Convolutional Neural Networks[Text Word]) OR (blockchain[Text Word]) AND (Environmental impact[Text Word]) OR (Greenhouse gases[Text Word]) OR (carbon footprint[Text Word]) AND (Gastroenterology[Text Word]) OR (digestive endoscopy[Text Word]) OR (capsule endoscopy[Text Word]) OR (gastrointestinal biopsy[Text Word]) OR (gastrointestinal lesion[Text Word]) OR (panendoscopy[Text Word]). The titles of the 704 articles recovered were manually screened, and the abstracts of those considered relevant were reviewed for the potential interest of their content.) In particular, we focus on situations when AI tools are employed in an attempt to enhance the diagnostic accuracy of these procedures. We address the ways in which this footprint can be considered to be at least partially offset or neutralized, contributing to the sustainability of these tools while ensuring their ethical status (e.g., lack of bias, etc. (Figure 1)). Interestingly, a recent life-cycle analysis of GI biopsy processing quantified the GHG emissions associated with these procedures and offered potential mitigating strategies [37]. Another novel technological advance that could offer solutions to these problems when implemented in healthcare settings is the relatively recently introduced blockchain technology. Whereas this technology also has a potential environmental impact, the possibilities that it can help mitigate the carbon footprint of AI tools are assessed.

## 2. Improved Efficiency of Gastrointestinal Examinations

It is becoming clear that the application of AI-based technologies does not come without a cost. Indeed, in the times in which we live, the environmental impact of all human activity is acquiring relevance, such that the carbon footprint associated with the implementation of AI-based technologies has become an important concern. These technologies place important demands on computational capacity, requiring the use of powerful GPUs and CPUs that consume a large amount of energy and that produce a considerable amount of heat, therefore requiring energy-demanding cooling systems. Moreover, they impose significant needs in terms of digital storage, also associated with important energy demands and a large carbon footprint. Furthermore, the environmental impact of obtaining the materials required to produce the devices used to run AI applications is also significant. These demands are all largely unavoidable, and while efforts can be made to dampen these, perhaps through carbon offsetting and gaining carbon credit, direct efforts should still be made to ensure AI solutions become more environmentally acceptable. Among the initiatives that aim to reduce the carbon footprint of AI tools, one area of interest has logically focused on minimizing the energy consumption of both the algorithms employed and the associated hardware. For example, the use of Spiking Neural Networks (SNNs) has been postulated as a promising energy efficient solution to more traditional ML models [38,39,40]. Based on the thresholds established in the brain that must be overcome before a neuron will fire and action potential, SNN models are designed to only process more relevant information, thereby reducing the energy consumption associated with the use of such models without any loss of accuracy. Similarly, neuromorphic computing is again inspired on the structure and function of the brain in an effort to make computational processes and data extraction more energy efficient. Similarly, efforts have been made to ensure more environmentally friendly data storage through the use of Spintronic devices [41,42,43].

Our interest here focuses on digestive endoscopy and, more specifically, on the application of AI solutions to CE. CE offers a series of advantages over traditional endoscopy [44,45]; for example, this procedure offers certain advantages when used to assess more inaccessible parts of the gastrointestinal (GI) tract (e.g., the small bowel) and when performing panendoscopy examinations [46,47,48,49]. Accessing more of the GI tract and in a more consistent manner obviously has important implications regarding the diagnostic accuracy of such procedures. As a result, CE can be used to evaluate conditions that otherwise present complications in reaching a clear diagnosis, such as chronic GI bleeding, angiodysplasia, GI tract tumours and especially SB tumours, small intestine mucosal damage, Crohn’s disease (CD), chronic iron-deficiency anaemia, GI polyposis or celiac disease [45,50]. Whereas enhancing access to all parts of the GI tract will generally have a positive effect on diagnostic accuracy, the increase in the number of images obtained and that must be analyzed by the specialist may also have a negative effect on their capacity to achieve a precise diagnosis. CE also paves the way for the analysis of endoscopic images or videos in real time [51,52,53,54], aiding clinicians in the more accurate and efficient identification of abnormalities and/or lesions. Finally, it is a procedure that can also overcome some of the disadvantages and adverse effects associated with standard colonoscopy procedures.

**Figure 2:**
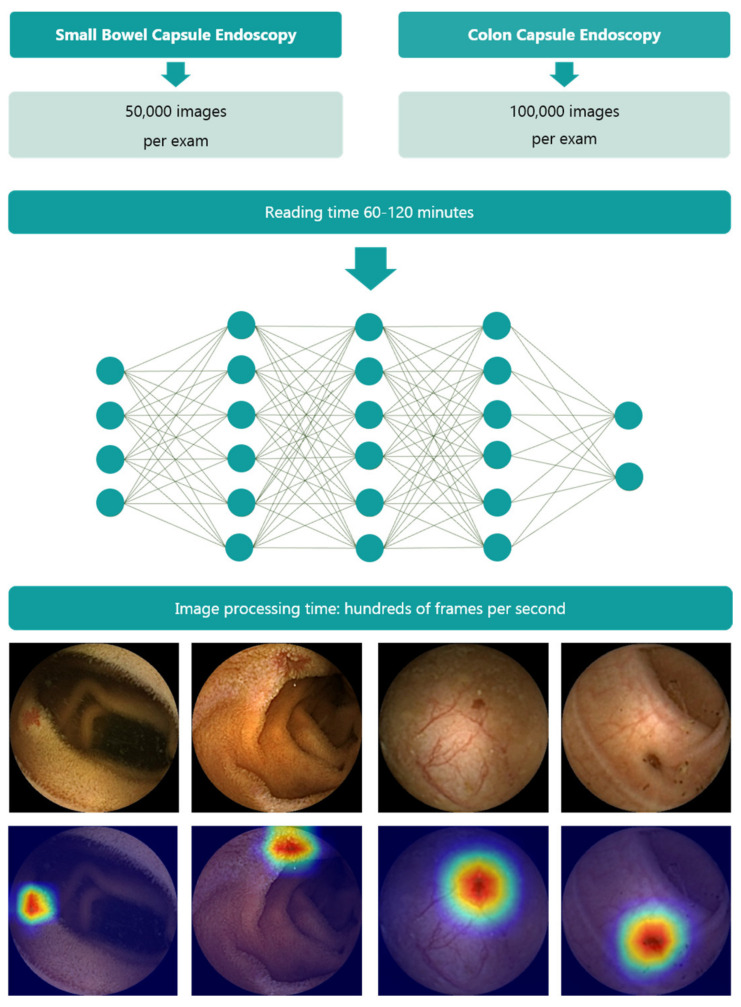
Capsule endoscopy examinations create a large number of frames (c.a. 100,000 frames per exam), with a reading time that limits the clinical application of the technology. Artificial intelligence overcomes this limitation as it is able to identify subtle lesions (see heatmaps) while exponentially decreasing the time required to read the exam.

At present, there is considerable interest in the design of AI-powered algorithms to be used in conjunction with CE, both to automate the reading of the images obtained by CE as well as to address associated issues (e.g., bowel cleanliness [21,55,56,57]). CE examinations may produce up to 100,000 images that a trained specialist requires up to 1.5 h to analyze. The time required by AI applications to read CE examinations may currently fall below 5 min, representing a more than notable improvement over standard procedures, with the ensuing cost savings [58,59]. This volume of images and the fatigue associated with their analysis may, to some extent, be implicated in the errors that could be made in achieving a diagnosis from these studies. Hence, the principal goal behind designing AI tools to assess CE examinations is to improve diagnostic accuracy and, if possible, achieve lower costs, providing benefits to both healthcare services and patients alike (Figure 2).

The development of AI systems that can predict the consequences of a particular histological feature will be important to screen patients, and these tools highlight the important role that CE can play in endoscopy diagnosis. Enhancing diagnostic accuracy while achieving time savings may be fundamental to the more widespread implementation of AI in conjunction with CE, particularly when considering this procedure as a valid screening and diagnostic procedure. However, the greatest potential benefits that could be achieved with CE examinations are likely to come in the future from the introduction of autonomous AI systems that function independently. In general, the AI tools currently under development for digestive health care mainly aim to be assistive, guiding the clinical decision-making process. Many of the initial efforts have centred on automating the detection and diagnosis of colorectal neoplasia, highlighting the potential of AI applied to CE to identify and characterise lesions in colorectal cancer screening [60,61,62]. The initial explorations of AI algorithms to identify colorectal neoplasia in CE images had relatively modest sensitivity [63]. However, to minimise the risk of missing clinically important diagnostic signs, it is necessary that such systems be very sensitive. Moreover, these tools must also display an adequate negative predictive value while retaining their specificity during the diagnostic work-up. We have since designed AI applications to automate the detection of protruding lesions in the colon through CE images [64], to accurately identify and diagnose lesions in the colon mucosa [20] and for the automated detection of blood/hematic residues in the colon lumen [65]. In each of these cases, the tools have achieved the specificity and sensitivity, as well as the predictive value, required to merit further validation towards their clinical implementation. Thus, the application of AI tools to CE appears to be able to potentially enhance the diagnostic accuracy of these procedures [21,55,56,57,58], reducing the number of times such examinations must be repeated and hence, minimizing the overall number of endoscopies performed. Moreover, this improvement in accuracy must also be considered longitudinally, given that AI tools are not subjected to fatigue as the day progresses or variations in performance that may be related to external factors that affect clinicians (health issues, momentary losses of concentration, etc.). Thus, any human errors that may be produced in the reading of CE can be avoided or severely reduced, with the associated economic benefits and the improvements to the carbon footprint involved. We consider introducing AI systems into the clinic will be a fundamental step to improve the diagnosis of GI tract disorders and colorectal neoplasia, addressing some of the limitations associated with CE (e.g., the long reading times) to lessen the burden on gastroenterologists.

As well as offering diagnostic support based on image analysis, by taking into account a patient’s medical history, AI tools can suggest optimal examination strategies, guide biopsy or resection decisions, or identify potential complications. This support can help clinicians make more efficient decisions, avoiding unnecessary actions that may increase the carbon footprint associated with gastroenterology departments. For example, the GHG emissions associated with GI biopsy processed annually in the US were estimated to be the equivalent of the emissions from 1200 passenger cars according to a recent life-cycle analysis [37]. Whereas mitigation strategies were proposed (e.g., through efficient use of biopsy jars and green equipment/supplies), it is clear that the most significant impact will be obtained by reducing the number of biopsies required, which could possibly be achieved through the use of AI tools that more accurately diagnose GI pathologies without the need to perform a biopsy.

**Figure 3:**
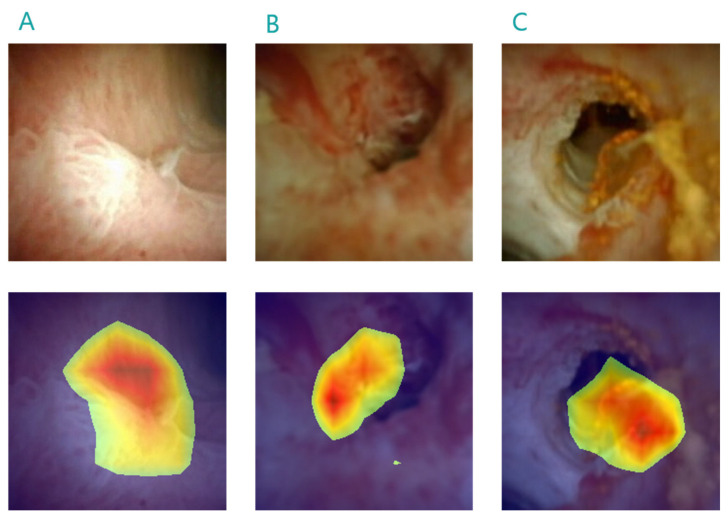
Heatmaps generated using a DL model during a digital cholangioscopy exam, identifying characteristics typically associated with malignancy, like papillary projections (**A**) and visible vessels (**B**,**C**). The heatmaps can guide endoscopic biopsies, increasing the cost-effectiveness of the examination and reducing the number of subsequent procedures.

A good example of the way in which AI tools can streamline the workload in digestive health care and reduce the need for biopsies is that of the automated detection of high-grade intraepithelial lesions (HSILs), lesions with a higher risk of progressing to anal squamous cell carcinomas (ASCC: [66]). Accurately detecting HSILs and distinguishing them from other lesions is critical, as HSIL detection requires active therapy to mitigate the progression of ASCC [67]. A DL model we designed and tested in a proof-of-concept study to detect HSIL lesions and distinguish them from less aggressive lesions (manuscript in preparation) is an example of how predicting histological types by AI, as opposed to biopsy, can reduce the number of interventions performed. A similar type of tool has also been designed to be used in cholangioscopy examinations (Figure 3). By reducing the number of procedures performed and, in particular, the number of unnecessary procedures carried out, AI can help conserve medical resources, including equipment, and reduce the consumables expenditure and energy required for each type of examination. This reduction in terms of consumables and energy will have a clear influence on the institutional carbon footprint, demands that will be additionally reduced by avoiding unnecessary displacement of patients and healthcare professionals to the clinical setting in which these examinations are carried out (Table 1). The enhanced precision in terms of lesion detection and localization, as well as the highlighting of suspicious areas, means that AI can help guide clinicians to focus only on relevant areas of the GI tube, avoiding unnecessary biopsies or interventions [11]. Such a targeted approach will reduce the number of biopsies examined, limiting the need to transport and further analyse tissue samples, with the associated burden on resources and waste generation. Indeed, transport is another of the most important influences on healthcare GHG emissions, including that of patients, providers and materials, and that of healthcare staff. Whereas reducing this part of the carbon footprint is unlikely to compensate fully for the emissions associated with the training of and use of a DL AI system, it will partially offset these carbon demands.

AI algorithms can also enhance quality assurance and assist procedures by reviewing endoscopic videos or images post-procedure. They can detect missed lesions or abnormalities, providing feedback to endoscopists for continuous improvement [11,58]. In addition, AI applications are being designed to evaluate the preparation and cleanliness of the gastrointestinal tract when performing these examinations [68,69,70]. This is one of the most important issues in defining the success of the examination, and if not adequately assessed, it is not clear whether the results of an examination can be trusted. Adequate preparation minimises missed or overlooked findings, confirming the validity of the examination. Hence, AI can reduce the need for repeat procedures or surveillance, thereby saving resources and lowering the environmental impact. When considering assistive and autonomous AI systems, the latter function independently and, as such, will clearly have a much stronger impact on the carbon emissions associated with CE examinations. Indeed, when referring to initial diabetic eye examinations, autonomous AI was proposed to reduce energy costs by around 80% relative to in-person eye examination [71]. Finally, AI tools may be able to mine our EMRs and identify illnesses that we may be prone to, favouring early detection with the important economic and health benefits associated with this [72,73,74]. Indeed, recognizing factors that could lead to the development of a specific disease could lead to adoption of measures and lifestyle changes that could avert the onset of such diseases. The clear enhancement of individual and population health that this would produce will be associated with a clear reduction in the healthcare-related carbon footprint, e.g., by reducing the consumption and use of medications, reduced testing, lowering the workload for healthcare employees (or by extension increasing the number if patients they can attend over the same period), fewer hospital visits and hospitalizations, etc.

The introduction of AI will also have an important impact on training and education in digestive medicine [75]. AI-based simulation platforms can be used to facilitate the training and education of endoscopists. These platforms provide realistic virtual environments for trainees to practice various procedures, reducing the need for live patient-based training [76]. Thus, by reducing the number of training procedures performed on actual patients, AI can help conserve resources and minimise the associated carbon footprint. Also, while the training of DL AI applications augments the energy demands [31,77] attributed to these applications this increase would most likely be considerably less than that associated with the training required to bring a novice endoscopist up to the functional level demonstrated by such DL applications.

AI applications can also be used to model and improve more administrative aspects of health care [78,79], such as the optimisation of workflow planning and the scheduling of medical interventions, the procedure types to which they are submitted and resource allocation, fine-tuning endoscopy unit workflows towards an optimum. Moreover, they can even be used to optimise electricity usage and the performance of heating/cooling systems [80,81]. By efficiently managing patient appointments and procedure sequencing, AI can reduce waiting times, minimise idle equipment and streamline resource utilization [79]. Such optimizations can help minimise energy consumption, reduce patient waiting times and displacements, and improve overall resource efficiency, with the consequent improvement in the environmental impact of digestive medicine as a whole. Moreover, when employing AI application in the cloud or accessing data in this way, the choice of low carbon data centres can also offset some of the carbon emissions that are unavoidable in health care [30].

## 3. Blockchain Technology and the Sustainability of Digestive Health Care

The introduction and implementation of blockchain technology in the healthcare environment can offer many advantages [82] and, not least, it may help decrease the carbon footprint of digestive health care in a variety of manners. Here, we shall touch on the ways that blockchain can facilitate sustainability in a clinical scenario and in relation to the use of CE in gastroenterology, particularly in association with the introduction of AI applications.

**Figure 4:**
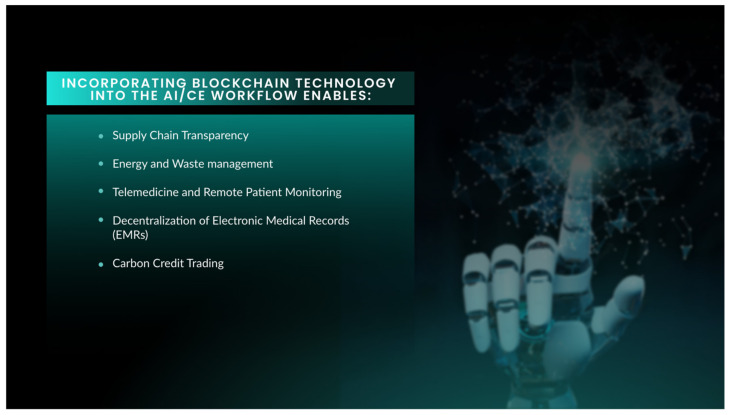
Blockchain technology offers a series of potential benefits when implemented in a healthcare environment, particularly when employed in conjunction with AI applications that have been designed to be applied with Capsule Endoscopy.

In terms of transparency in the supply chain, blockchain technology can provide end-to-end visibility of the supply chain for the medical devices, pharmaceuticals and consumables used in digestive health care [83]. By tracking the origin, manufacturing processes, transportation and disposal of these products, blockchain can help minimise the carbon footprint associated with the procurement of these supplies, overcoming inefficiencies [84]. Likewise, waste management can be similarly optimised, streamlining this by tracking the disposal and recycling of medical waste generated during digestive procedures [85]. By ensuring proper disposal practices and optimizing recycling efforts, blockchain can help minimise the environmental impact of these procedures and promote sustainable waste management practices (Figure 4).

As we have seen, energy management is a fundamental issue in relation to the sustainability and optimization of health care in general, blockchain can track and optimise energy usage in healthcare facilities. Smart contracts promoted through blockchain can automate energy consumption based on demand, optimise energy distribution and incentivise more energy-efficient practices, leading to reduced energy consumption and lower carbon emissions [86]. In this regard, blockchain can also drive the creation and trading of carbon credits within the healthcare industry. By tokenizing carbon credits on a blockchain platform, healthcare providers can track and exchange credits based on their efforts to reduce their carbon footprint [87]. This would incentivize environmentally friendly practices and encourage carbon neutrality in digestive health care.

Blockchain can enable secure and interoperable handling of EMRs, allowing patients and healthcare providers to access and share medical information seamlessly [88,89]. By eliminating the need for paper-based records and their transportation, and of redundant tests, blockchain-based EMRs improve data accuracy, reduce administrative waste and decrease the carbon footprint associated with physical record keeping while ensuring data privacy and accessibility. blockchain can also support secure and decentralized storage of patient health data for telemedicine and remote patient monitoring applications [90,91,92,93]. This is an important issue as it paves the way to access data centres with smaller carbon footprints. Given the reduced travel and displacement associated with telemedicine, both of patients and healthcare providers, it is generally considered to reduce emissions [94,95]. Similarly, blockchain can facilitate secure and transparent sharing of research data among healthcare institutions. By providing a decentralized and immutable ledger, blockchain allows researchers to collaborate on large-scale studies without duplicating efforts. This reduces redundant data collection, minimises resource consumption and promotes efficient research practices.

Finally, blockchain-based incentive programs can motivate patients to adopt healthier lifestyles and participate in preventive care [96,97]. By rewarding patients with blockchain-based tokens or digital assets for achieving health goals, patients are encouraged to engage in proactive measures that reduce the need for invasive digestive healthcare procedures, consequently lowering the associated carbon footprint.

## 4. Conclusions

The environmental impact of AI solutions is largely associated with the energy demands associated with these tools and the impact attributed to the manufacture of the devices required to run them. When used in digestive health care, the impact of these tools will be added to the carbon footprint associated with health care in general, which is not inconsiderable. However, while certain advances are being made in reducing the impact associated with AI (e.g., neuromorphic modelling), perhaps carbon offsetting in the digestive healthcare scenario must be considered in full when defining the sustainability of these applications. AI tools can improve the diagnostic accuracy of CE examinations and the time required to analyse these. By enhancing the diagnostic accuracy of these procedures, the number of repeat examinations and the need for additional tests will be reduced, as will the equipment and consumables used, the dedication of healthcare professionals and all the related transport costs. Moreover, we also highlighted the benefits of introducing blockchain into the healthcare environment, particularly when employed in conjunction with AI applications. To date, no direct comparisons have been made of the environmental impact of these novel approaches and the current gold standard techniques employed to examine the GI tract. Whereas these are not necessarily easy to perform, such studies would provide evidence of the environmental benefits that can be gained by introducing these novel procedures, should they exist, as well as identifying the aspects of both the novel and traditional approaches that are most damaging to the environment and that could be targeted to reduce their impact where possible. Nevertheless, in terms of sustainability and the carbon footprint, we believe that the incorporation of novel AI tools in conjunction with CE, and the implementation of other technologies into digestive health care, will drive important improvements in this area over the forthcoming years.

## Figures and Tables

**Table 1:** The environmental benefits of using minimally invasive Capsule (Pan)Endoscopy versus traditional upper endoscopy and colonoscopy.

Feature	Benefits
Non-invasive procedure	As a non-invasive procedure, CE eliminates the need for sedation or anesthesia, reducing the associated energy consumption and carbon emissions.
Simplified examination requiring less equipment	CE eliminates the need for complex, bulky and specialized equipment (e.g., endoscopes, light sources and video processors). Using a small ingestible capsule with an embedded camera, CE significantly reduces the energy consumption, maintenance and disposal of traditional endoscopy material, thereby minimising the environmental impact of the procedure.
Fewer resources consumed	CE avoids the use of consumables typically required in conventional endoscopy procedures, like biopsy forceps and cleaning brushes. Lower demands on resources translates into less material waste and energy used in manufacturing, as well as fewer demands for sterilization, leading to a smaller carbon footprint.
Procedure time	CE is generally quicker than traditional endoscopy and patients typically recover faster, minimising waiting times and the resource use associated with keeping patients in dedicated endoscopy units for longer periods.
Improved patient compliance	CE is more patient-friendly, which enhances compliance with screening protocols, avoiding unnecessary examinations that further tighten the carbon footprint.
Targeted examination	CE may target specific areas of the GI tract, avoiding unnecessary full-length examinations, reducing procedure time and resource consumption, as well as the associated carbon emissions.
Enhanced diagnostic accuracy	CE produces high-resolution images, improving diagnostic accuracy and reducing missed findings, unnecessary interventions and the need for further confirmatory procedures, enhancing resource use.
Remote viewing and consultations	CE images and videos can be viewed and analyzed remotely by specialists, reducing transport-related GHG emissions by eliminating the need to transport patients or their records, favoring more efficient consultations that save time, energy and resources.

## Data Availability

Not applicable.
